# Genome-wide analysis of the *ABC* gene family in almond and functional predictions during flower development, freezing stress, and salt stress

**DOI:** 10.1186/s12870-023-04698-7

**Published:** 2024-01-02

**Authors:** Dongdong Zhang, Zhenfan Yu, Bin Zeng, Xingyue Liu

**Affiliations:** College of Horticulture, Xinjiang Agricultural University, Urumqi, 830000 China

**Keywords:** *Prunus dulcis*, *ABC* gene family, Fluorescence quantification, Flower development, Freezing stress, Salt stress

## Abstract

ABC (ATP-binding cassette) transporter proteins are one of the most extensive protein families known to date and are ubiquitously found in animals, plants, and microorganisms. *ABCs* have a variety of functions, such as plant tissue development regulation, hormone transport, and biotic and abiotic stress resistance. However, the gene characterization and function of the *ABC* gene family in almond (*Prunus dulcis*) have not been thoroughly studied. In this study, we identified 117 *PdABC* genes using the whole genome of ‘Wanfeng’ almond obtained by sequencing and explored their protein characterization. The *PdABC* family members were classified into eight subfamilies. The members of the same subfamily had conserved motifs but poorly conserved numbers of exons and introns and were unevenly distributed among the eight subfamilies and on the eight chromosomes. Expression patterns showed that *PdABC* family members were significantly differentially expressed during almond development, dormant freezing stress, and salt stress. We found that *PdABC59* and *PdABC77* had extremely high expression levels in pollen. *PdABC63* and *PdABC64* had high expression levels during almond petal development and multiple stages of flower development. *PdABC98* was highly expressed in annual dormant branches after six temperature-freezing stress treatments. *PdABC29*, *PdABC69*, and *PdABC98* were highly expressed under different concentrations of salt stress. This study preliminarily investigated the expression characteristics of *ABC* genes in different tissues of almond during flower development, freezing stress and salt stress, and the results will provide a reference for further in-depth research and breeding of almond in the future.

**Supplementary Information:**

The online version contains supplementary material available at 10.1186/s12870-023-04698-7.

## Introduction

ABC (ATP-binding cassette) transporter proteins comprise one of the largest families of proteins discovered to date and are widely found in animals, plants, and microorganisms [1]. ABC transporter proteins can take up a wide range of substances, such as inorganic ions, nutrients, amino acids, and organic matter, to maintain the normal biological activities of the organism [2]. ABC transporter proteins have a highly conserved nucleotide-binding domain (NBD) and transmembrane domain (TMD) with five or six helices [3]. These transporter proteins are categorized into three types of structures: holomolecules, semimolecules, and soluble proteins. Holomolecular transporter proteins contain two NBDs and TMDs, semimolecular transporter proteins contain one NBD and TMD, and soluble proteins have only an NBD and no TMD [4]. Among them, the TMD functions as a recognition substrate to transport substances across the membrane, and the NBD is located on the inner side of the cell membrane, including the conserved Walker A and Walker B sequences and the less conserved LSGGQ motif [5].

To date, ABC transporter proteins have been extensively studied in various species, including humans, insects, microorganisms, and plants [6–9]. According to related research, plant *ABC* members are typically divided into eight subfamilies: ABCA, ABCB, ABCC, ABCD, ABCE, ABCF, ABCG, and ABCI, with no ABCH subfamily identified in plants [10]. Currently, the most extensively studied subfamilies in different species are the ABCB, ABCC, and ABCG subfamilies [11]. Among these subfamilies, the ABCA (*AOH*, ABC1 homolog), ABCB (*MDR*, multidrug resistance protein), ABCC (*MRP*, multidrug resistance-associated protein), and ABCG (*PDR*, pleiotropic drug resistance protein) subfamilies are classified as full-transporter ABC proteins, while the ABCA (*ATH*, ABC2 homolog), ABCB (*ATM*, ABC transporter of the mitochondrion), ABCB (*TAP*, transporter associated with antigen processing), ABCD (*PMP*, peroxisomal membrane protein 3), and ABCG (*WBC*, white‒brown complex protein) subfamilies are categorized as half-transporter ABC proteins. The ABCE (*RLI*, RNase L inhibitor), ABCF (*GCN*, general control nonrepressible protein), ABCI (*SMC*, structural maintenance of chromosome protein), and ABCI (*NAP*, nonintrinsic ABC proteins) subfamilies are soluble ABC transport proteins [12]. The ABCA subfamily includes both full-transporter AOH and half-transporter ATH types. For example, *A. thaliana* contains one AOH and 16 ATH type transporter proteins [13]. Notably, *AOH*-type genes have only been found in dicotyledonous plants such as *Arabidopsis* and grapes and are absent in monocotyledonous plants such as maize [14, 15]. The ABCB subfamily includes three classes of transporter proteins: MDR, ATM, and TAP. In *Arabidopsis*, 29 members of this subfamily have been identified [13]. Research has revealed that plant ABCB subfamily members are primarily involved in the regulation and transport of auxins (such as *AtPGP1* and *OsABCB14*) and participate in ion transport and resistance to heavy metal stress (including *OsABCB25* and *OsABCB23*) [16–19]. The ABCC subfamily encodes MRP-type transporter proteins, which are associated with transport and metabolic functions in plants. For instance, *AtABCC4* is involved in folate transport, while *VvABCC1* and *AtABCC2* participate in anthocyanin transport [20–22]. Additionally, members of this subfamily in plants have detoxification functions. *AtMRP1* and *AtMRP2*, among others, are involved in resistance to heavy metals [23, 24]. The ABCD subfamily encodes PMP-type transporter proteins, with *AtPMP2* shown to inhibit seed germination [25]. The ABCG subfamily includes two classes of transporter proteins: PDR and WBC. This is the largest subfamily with members that have diverse functions, including regulating plant terpenoids, alkaloids, lipids, and volatile aromatic compounds [26–28]. The ABCE, ABCF, and ABCI subfamilies lack transmembrane domains and do not exhibit specific transport characteristics.

Almond, belonging to the genus *Prunus* in the family *Rosaceae*, is a medium-sized tree or shrub. Its kernel is highly nutritious and medicinally important, making it widely cultivated and utilized in various countries worldwide. Xinjiang is a major cultivation area of almonds in China, covering an area of approximately 66,000 hectares, and almonds are an important economic fruit tree in this region. However, a major challenge in almond production in Xinjiang is the low pollen activity and reduced fruit set due to self-incompatibility, which severely hinders the development of the almond industry. Therefore, it is of significance to explore the potential functions of *ABC* genes in different floral organs of almond. Additionally, there are other common issues that impact the almond industry, such as early flowering of native almond varieties in Xinjiang, making them vulnerable to late spring frosts, and freezing damage to floral organs in extremely low-temperature conditions during the winter dormancy period. This region also faces the challenge of severe soil salinity and alkalinity, which limits the development of the almond industry. To gain insight into the characteristics of *ABC* gene family members in almond, we conducted a comprehensive analysis based on ‘Wanfeng’ almond whole-genome data. We identified and characterized *ABC* gene family members and performed bioinformatics analyses to explore protein physicochemical properties, phylogenetic tree construction and classification, conserved motifs, gene structures, gene chromosomal localization, gene duplication types, *cis*-acting elements, and expression patterns. Furthermore, we conducted fluorescent quantitative expression analysis of 10 *PdABC* genes in various floral organs of almonds across six different stages of flower development under a gradient of six temperatures to simulate freezing stress conditions during the dormancy period and subjected them to three different salt stress treatments. The aim of this study was to explore the potential functions of ‘Wanfeng’ almond *ABC* gene family members in floral organ development and stress responses, thereby providing valuable insights for future research related to almonds.

## Materials and methods

### Identification of almond *ABC* family members

The whole genome data of *Prunus dulcis* ‘Wanfeng’ were used for the ABC gene family analysis [29]. The Pfam database (http://pfam.xfam.org/) was employed to download hidden Markov models (HMMs) for four ABC transporter domains: ABC transporter domain (PF00005), ABC transporter transmembrane region domain (PF00664), ABC 3 transport family (PF00950), and ABC 3 transport family (PF00950). The HMMER tool was used to search and compare the almond whole-genome protein sequences with the four ABC transporter HMMs, retaining proteins with e-value ≤ 1e^−5^ [30]. The BLASTP program was employed to compare almond whole-genome protein sequences with *Arabidopsis* ABC member protein sequences, retaining proteins with e-value ≤ 1e^−5^. Combining the HMMER and BLASTP results, duplicate sequences were removed, and ABC domains were validated using the NCBI-CDD tool (https://www.ncbi.nlm.nih.gov/cdd/). A total of 117 *ABC* genes were identified in the almond genome. Protein physicochemical properties were determined using ExPASy (http://web.ExPASy.org/protparam/), and subcellular localization was predicted using WoLF PSORT II (https://www.genscript.com/wolf-psort.html?src=leftbar) [31, 32].

### Construction and classification of phylogenetic trees

A total of 129 protein sequences of *A. thaliana* ABC family members were downloaded from the UniProt database (https://www.uniprot.org/). MUSCLE multiple sequence alignment was performed using MEGA X. The maximum likelihood method was used to construct phylogenetic trees with 1,000 bootstrap replications, Poisson correction model, and pairwise deletion [33]. Further phylogenetic analysis included the examination of *ABC* members associated with flower organ development in *A. thaliana*, *Petunia hybrida*, *Medicago truncatula*, *Vitis vinifera*, and *Malus domestica*.

### Motifs, domains, and gene structures

The MEME online tool (http://meme-suite.org/tools/meme) was used to identify motifs in *PdABC* family member protein sequences [34]. The NCBI CDD online tool (https://www.ncbi.nlm.nih.gov/cdd/) was used to retrieve information on the location and number of ABC domains in *PdABC* family member protein sequences [35]. Gene structure information, including exon and intron positions and numbers, was extracted from the almond genome GFF (General Feature Format) data. TBtools was employed for clustering and visualization of *PdABC* family members phylogenetic tree, motifs, domains, and gene structures [36].

### Gene mapping, collinearity, and Ka/Ks analysis

The TBtools was used to map the positions of *PdABC* members on chromosomes. The MCScanX tool was used for segmental and tandem duplication gene analysis, and the Circos tool was used for drawing chromosome collinearity diagrams [37, 38]. *ABC* collinearity gene analysis was further conducted among *P*.*dulcis*, *A*.*thaliana*, *Oryza sativa*, *M*.*domestica* and *Prunus persica*. The Ka/Ks Calculator tool was used to calculate Ka (Nonsynonymous substitution rate), Ks (Synonymous substitution rate), and Ka/Ks values for duplicated genes [39].

### *Cis*-acting element annotation

Upstream 2000 bp sequences of the 117 *PdABC* genes were extracted using TBtools. The PlantCARE online tool (http://bioinformatics.psb.ugent.be/webtools/plantcare/html/) was employed for *cis*-acting element annotation in the promoter regions [40]. *Cis*-acting elements associated with abiotic and biotic stresses, phytohormone responsiveness, and plant growth and development were selected and analyzed [41]. *Cis*-acting element distribution maps of *PdABC* members were generated using TBtools.

### Expression patterns

FPKM (Fragments Per Kilobase of exon model per Million mapped fragments) values were obtained from transcriptome data for ‘Wanfeng’ almond, which included six flower developmental stages (GSA Number: CRA007615), a gradient of six freezing temperature stress conditions for dormancy (GSA Number: CRA007323), and salt stress treatments for ‘Amanisha’ almond (GSA Number: CRA013033). Transcriptome data are available through the CNCB database (https://www.cncb.ac.cn/).

### Protein‒protein interaction network construction

Protein sequences of almond PdABC family members were compared in the STRING database (https://string-db.org/) to predict protein‒protein interactions among PdABC members. The Cytoscape tool was used to visualize the protein interaction network.

### Fluorescence quantification

We selected three six-year-old ‘Wanfeng’ almond trees with good growth as material collection objects through the almond resource orchard of the state-owned second forest farm in Yarkand, Kashgar, Xinjiang, China. The three trees corresponded to three biological replicates. Various ‘Wanfeng’ almond tissues (leaves, sepals, petals, pollen, and pistils), flowers at six stages of development [29], dormant one-year-old branches under freezing stress (CK (—5 ℃), —10 ℃, —15 ℃, —20 ℃, —25 ℃, and —30 ℃) at different temperatures [42]. In August 2022, ‘Amanisha’ almond seeds were collected through the almond resource orchard, and dormancy was artificially broken in the laboratory in November 2022. The seeds were placed in seedling trays with soil and cultured in a solar greenhouse. By May 2023, 12 plants were selected to grow well. The seedlings (six months) were watered with NaCl solutions of four concentrations: CK (0), 50, 100, and 150 mM. Three columns of seedlings were treated with each solution concentration as three biological replicates, and the leaves were collected after 24 h.

**Fig. 1:**
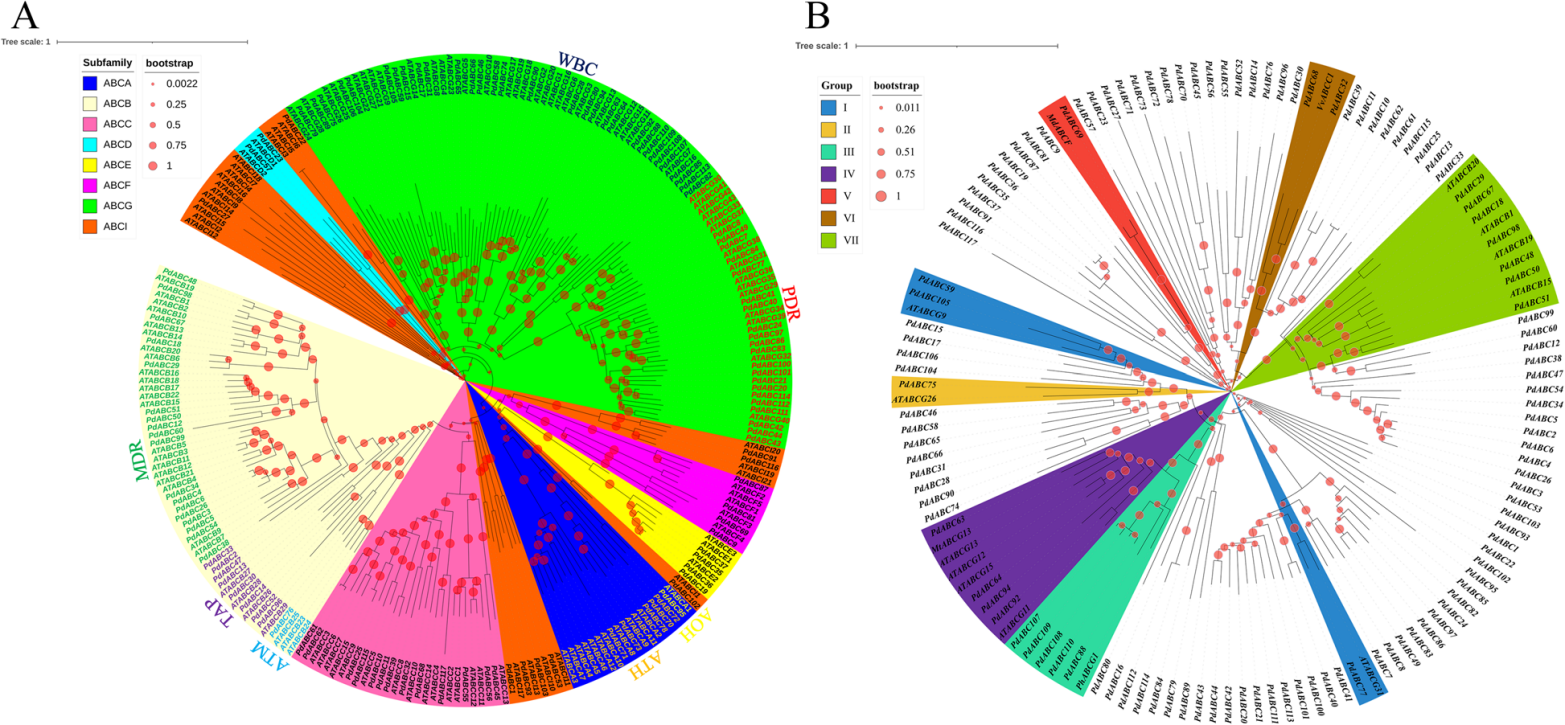
Phylogenetic Tree of *PdABC* Family Members. **A** Phylogenetic tree of Wanfeng almond and *Arabidopsis* ABC family members. Each colored region represents a subfamily, with yellow font denoting AOH, orange font denoting ATH, light blue font denoting ATM, purple font denoting TAP, green font denoting MDR, red font denoting PDR, and dark blue font denoting WBC. **B** Phylogenetic tree of *PdABC* family members in relation to *ABC* genes associated with flower organ development in five species: *A. thaliana*, *P. hybrida*, *M. truncatula*, *V*. *vinifera*, and *M. domestica*. Each coloured region represents a group

In this study, we primarily focused on *PdABC* genes related to almond pollen development. Therefore, based on the phylogenetic clustering results shown in Fig. 1B, we selected 10 *PdABC* genes closely associated with pollen development for fluorescent quantitative analysis. Three independent biological replicates containing three independent plants were used for qRT‒PCR. The qRT‒PCR primers of the selected *PdABC* genes were designed by Primer Premier 5 (Table S1). The fluorescent quantitative analysis process followed the same methodology as previously described by our research team [43]. The obtained cycle threshold (CT) values were quantitatively analyzed by the 2^-△△Ct method (Table S2) [44].

## Results

### Characteristics of *PdABC* family members

We identified 117 *ABC* genes in the ‘Wanfeng’ almond genome, and NCBI-CDD database verification confirmed the presence of an ABC domain in each protein sequence of each gene. These 117 *ABC* genes were renamed *PdABC1* to *PdABC117* based on their chromosomal and positional distribution. The physicochemical properties of PdABC family members exhibited significant variation (Table S3). Notably, the number of amino acids in these members ranged from 108 aa (amino acids) (PdABC13) to 2837 aa (PdABC20). The molecular weight of PdABC family members ranged from 11,621.36 Da (Dalton) to 319,923.26 Da. Members were categorized into four groups by molecular weight: 10,000 Da to 49,999 Da, 50,000 Da to 99,999 Da, 100,000 Da to 149,999 Da, and over 150,000 Da, comprising 11, 50, 23, and 33 members, respectively. The isoelectric point of PdABC members ranged from 5.29 to 9.67, with 27 members having isoelectric point values less than 7 and 90 members having isoelectric point values greater than 7. Protein hydrophilicity analysis revealed that 37 members were hydrophilic proteins, while 80 members were hydrophobic proteins. Subcellular localization prediction placed members in five subcellular locations: nucleus, cytoplasm, plasma membrane, chloroplasts, and mitochondria, with 7, 19, 79, 10, and 2 members, respectively.

### Phylogenetic analysis of *PdABC* family members

We constructed a maximum likelihood phylogenetic tree using protein sequences of PdABC (117) and ATABC (129) members. The clustering of *PdABC* members was further explored based on the subfamily classification of *ATABC* members (Fig. 1A). We categorized *PdABC* members into eight subfamilies: ABCA, ABCB, ABCC, ABCD, ABCE, ABCF, ABCG, and ABCI, with each subfamily having 6, 27, 13, 2, 4, 4, 52, and 9 members, respectively (Table S3). Based on the clustering results of *ATABC* members, we further classified *PdABC* members into seven subcategories: ABCA (AOH), ABCA (ATH), ABCB (ATM), ABCB (TAP), ABCB (MDR), ABCG (PDR), and ABCG (WBC), with each category containing 1, 5, 1, 18, 8, 21, and 31 members, respectively. A comparison of *ABC* member numbers in nine different species revealed a high level of conservation in the distribution of *ABC* members across the eight subfamilies (Table S4). Notably, the number of *ABC* members in Wanfeng almond was similar to that in *Prunus avium*. The trends in ABC member distribution across the eight subfamilies in *P*. *avium*, *Prunus mume*, *P. persica*, and *P. dulcis* were similar. Additionally, based on the clustering of PdABCs with *ABC* genes associated with flower organ development in five species, the phylogenetic tree was divided into seven groups, labeled I to VII. Each group consisted of 3 (*PdABC59*/*77*/*105*), 1 (*PdABC75*), 5 (*PdABC88*/*107*/*108*/*109*/*110*), 4 (*PdABC63*/*64*/*92*/*94*), 1 (*PdABC69*), 2 (*PdABC32*/*68*), and 7 (*PdABC18*/*29*/*48*/*50*/*51*/*67*/*98*) members. It is hypothesized that these genes may play specific roles in almond flower organ development (Fig. 1B, Table S5).

### Analysis of conserved motifs, domains, and gene structures of *PdABC* family members

**Fig. 2:**
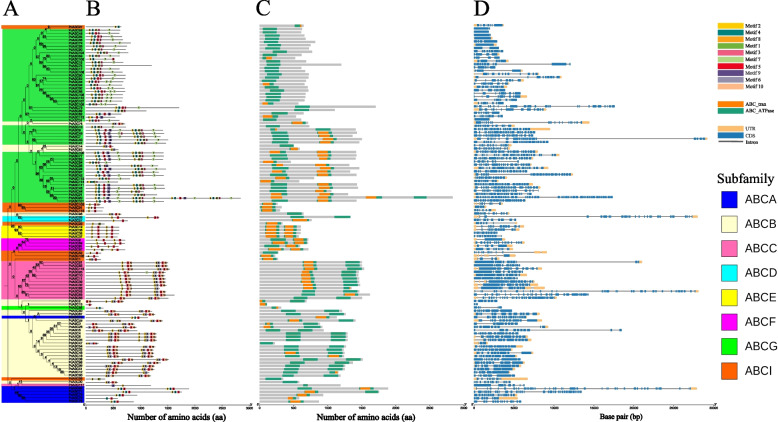
Conserved motifs and gene structures of *PdABC* family members. **A** Maximum likelihood phylogenetic tree of *PdABC* family members. **B** Conserved protein motifs. **C** ABC protein domains. **D** Gene structure

Based on the MEME tool, we annotated 10 motifs within the 117 *PdABC* family members and identified each motif sequence in the NCBI-CDD database. Notably, Motif 1, Motif 2, Motif 4, Motif 5, Motif 6, and Motif 10 correspond to the Walker A/Walker B motifs (ABC), while Motif 3, Motif 7, and Motif 9 are related to ABC transporter G family members, and Motif 8 represents the ABC-type lipoprotein export system (Fig. S1). The phylogenetic tree clustering of *PdABC* family members showed that members within the same subfamily exhibit a high degree of motif conservation (Fig. 2A, B). Specifically, ABCA subfamily members consist of a single type of Motif 2, Motif 4, and Motif 5. Members of the ABCB subfamily predominantly possess two regions, Motif 2, Motif 6, Motif 5, and Motif 10. ABCC subfamily members primarily contain two regions: Motif 2, Motif 5, Motif 10 or Motif 2, Motif 6, Motif 5, and Motif 10. Members of the ABCD, ABCE, ABCF, and ABCI subfamilies consist of Motif 2 and Motif 5. In contrast, ABCG subfamily members exhibit eight different motif types, namely, Motif 1, Motif 2, Motif 3, Motif 4, Motif 5, Motif 7, Motif 8, and Motif 9. Notably, the ABCG subfamily features three unique ABC transporter G motifs, Motif 9, Motif 3, and Motif 7, arranged sequentially. In addition, the distribution characteristics and quantity of PdABC protein domains are consistent with those of conserved motifs, further indicating the high accuracy of subfamily clustering corresponding to *PdABC* family members (Fig. 2C).

In terms of gene structure, the number of exons in *PdABC* family members ranged from 1 to 48, and the number of introns varied from 0 to 47, indicating a wide distribution (Fig. 2D, Table S3). The conservation of protein sequence lengths differs between different subfamilies. The sequence lengths of the three subfamilies ABCA, ABCB and ABCG are poorly conserved, with 3 (3), 16 (11) and 21 (31) members having sequence lengths greater than 1000 (less than 1000) amino acids, respectively. Notably, the sequence length of most members of the two categories ABCB (MDR) and ABCG (PDR) is more than 1000 amino acids, and they are highly conserved. The sequence lengths of the four subfamilies ABCC, ABCE, ABCF and ABCI are highly conserved, with average lengths of 1461, 597, 685 and 332 amino acids, respectively. There are only 2 members of the ABCD subfamily, which are 1340 and 764 amino acids. Similarly, the exon numbers of *PdABC* members among the eight subfamilies also varied greatly. The exon numbers of the six members of the ABCA subfamily are 8, 14, 16, 18, 34 and 40. The number of exons of the ABCB subfamily members is less than 20, with 1, 1, 2, 3, 1, 3, 6, 1, 5, 2, 1 and 1 members containing 2, 3, 6, and 7, respectively. Exons 8, 9, 10, 11, 12, 15, 17 and 18. The ABCC subfamily has 1, 1, 5, 2, 1, 1, 1 and 1 members with 7, 8, 10, 11, 12, 26, 28 and 36 exons, respectively. The exon numbers of the two members of the ABCD subfamily are 10 and 25, respectively. The number of exons among the four members of the ABCE subfamily is 11. The exon numbers of the four members of the ABCF subfamily are 1, 5, 9 and 18. The exon numbers of the ABCG subfamily members are relatively wide, with 4 members having 1 exon and 10, 12, 9 and 16 members having exon numbers ranging from 2 to 5, 7 to 9, 10 to 19, and 20 to 25, respectively, and one member has 48 exons. The ABCI subfamily has one exon, 6 exons with 3 to 8 exons, and 2 exons with 10 exons. Notably, in addition to the highly conserved exon number among members of the ABCE subfamily, the number of exons varies greatly among other subfamily members, which is one of the reasons for the functional diversity of members of the same subfamily. There is a strong correlation between the number of exons and the length of the protein of most PdABC members. The longer the protein sequence is, the greater the number of exons.

### *PdABC* family member chromosome location, collinearity, and Ka/Ks analyses

**Fig. 3:**
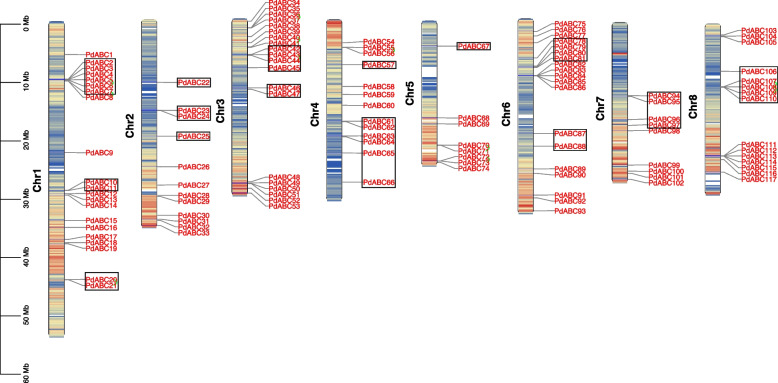
Chromosome locations of *PdABC* family members. The left scale represents chromosome lengths. Black squares represent *PdABC* genes located in low gene density regions, and green lines indicate tandem repeat gene pairs

The 117 *PdABC* family members are unevenly distributed across eight chromosomes, with chromosomes 1 to 8 carrying 21, 12, 20, 13, 8, 19, 9, and 15 genes, respectively. To calculate the gene density on each chromosome, we used a genetic distance of 200 kb and represented it in Fig. 3 as a color gradient from blue (low gene density) to red (high gene density). Blank regions indicate a lack of gene distribution information (Fig. 3; Table S6). Notably, 36.75% (43) of the *PdABC* family members on chromosomes 1 to 8 are located in low gene density regions. Similar to other plant *ABC* family studies, there is no significant correlation between chromosome length and the number of *PdABC* genes.

**Fig. 4:**
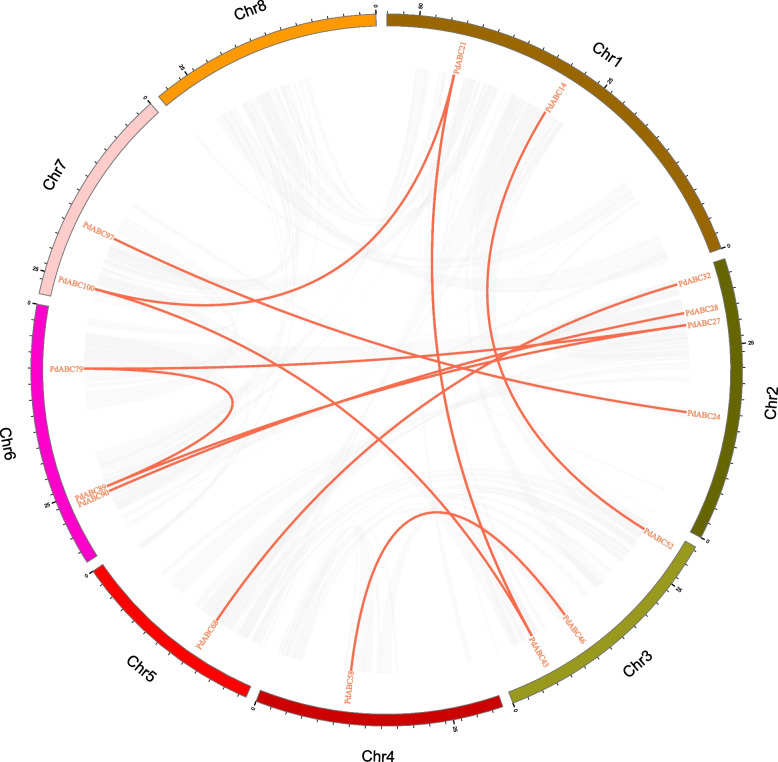
Interchromosomal relationships of *PdABC* genes in the almond genome. The red lines connect duplicated *PdABC* gene pairs

We identified 12 pairs of segmental duplicate genes and 11 pairs of tandem duplicate genes in the *PdABC* family members (Table S7). Notably, the ABCG (PDR) subfamily has 8 pairs of segmental duplicate genes and 6 pairs of tandem duplicate genes. The 12 pairs of segmental duplicate genes were distributed on all seven chromosomes except chromosome 8, with chromosomes 1 to 7 carrying 2, 4, 3, 1, 1, 3, and 2 genes, respectively (Fig. 4). The 11 pairs of tandem duplicate genes are distributed on five chromosomes, with chromosomes 1, 3, 4, 5, and 8 carrying 3, 3, 1, 2, and 2 genes, respectively. Additionally, four pairs of tandem duplicate genes, *PdABC5*/*PdABC6*, *PdABC43*/*PdABC44*, *PdABC107*/*PdABC108*, and *PdABC108*/*PdABC109*, are located in regions with low gene density on chromosomes (Fig. 3). Furthermore, the Ka/Ks values for the duplicated *PdABC* gene pairs are all less than 1, indicating significant purifying selection pressure during the evolution of *PdABC* family members (Table S8).

**Fig. 5:**
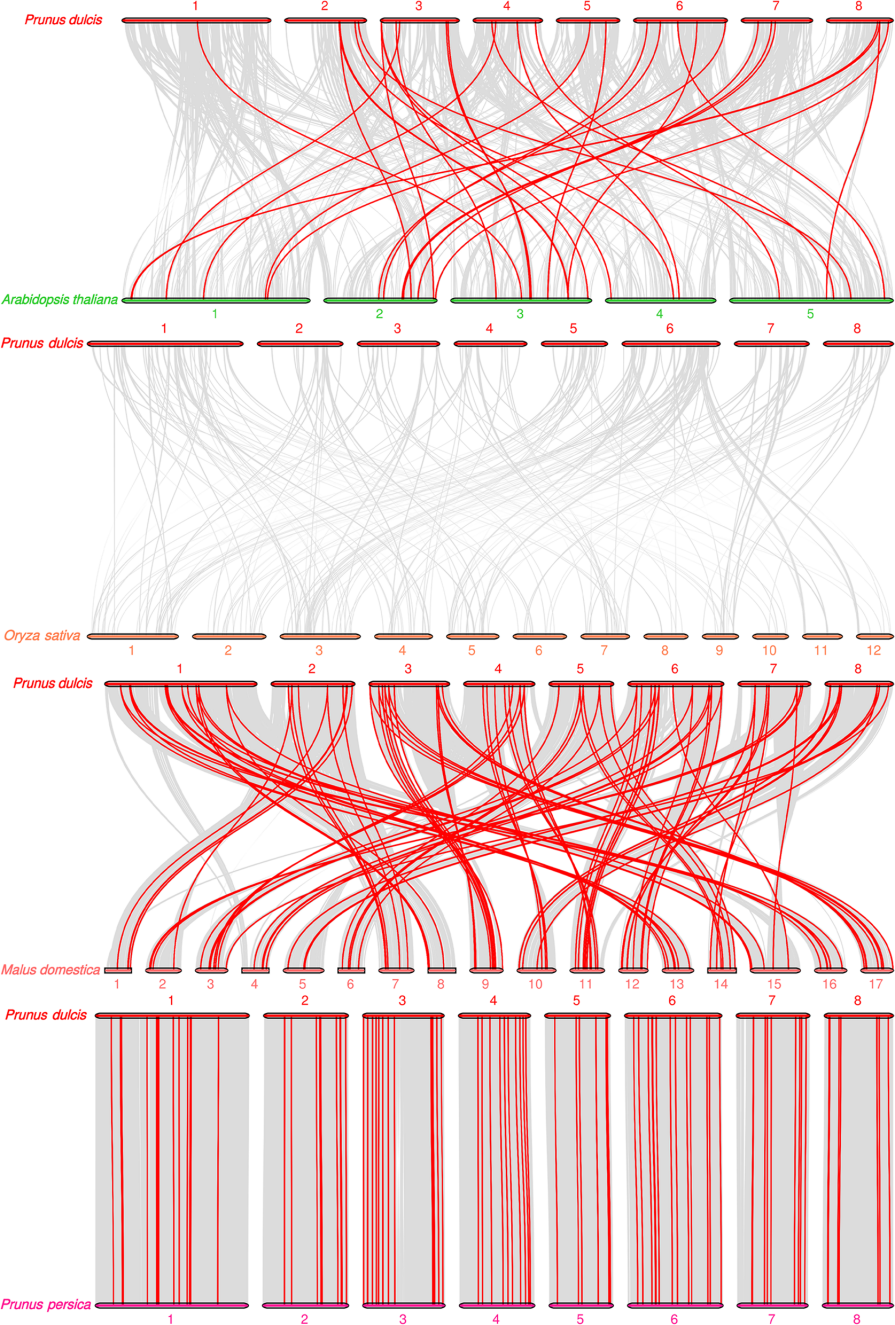
Synteny analysis of almond with *ABC* family members from four species. Each horizontal line represents a chromosome, and the numbers represent chromosome numbers. The red lines indicate collinear gene pairs

To investigate the evolution of *PdABC* family members, we assessed the collinearity of *ABC* family member genes in *P. dulcis* with those of four other species: *A. thaliana*, *O. sativa*, *M. domestica*, and *P. persica* (Fig. 5). The results show that almond has 32, 0, 108, and 94 collinear gene pairs with *A. thaliana*, *O. sativa*, *M. domestica*, and *P. persica ABC* family members, respectively (Table S7). In *A. thaliana* and *M. domestica*, some *PdABC* genes have collinear relationships with two or more *ABC* members, suggesting their important roles during the evolution of the *PdABC* gene family. The collinear gene pairs between *P. dulcis* and *P. persica* are generally one-to-one, indicating a high degree of conservation. Notably, there were no collinear gene pairs between almond and rice, suggesting a strong separation between monocots and dicots in the evolution of *ABC* genes. Furthermore, the Ka/Ks values for collinear gene pairs between *P. dulcis* and *A. thaliana*, *M. domestica*, and *P. persica* are all less than 1, indicating the influence of purifying selection pressure on *PdABC* family members during evolution.

### Upstream *Cis*-acting element analysis of *PdABC* family members

**Fig. 6:**
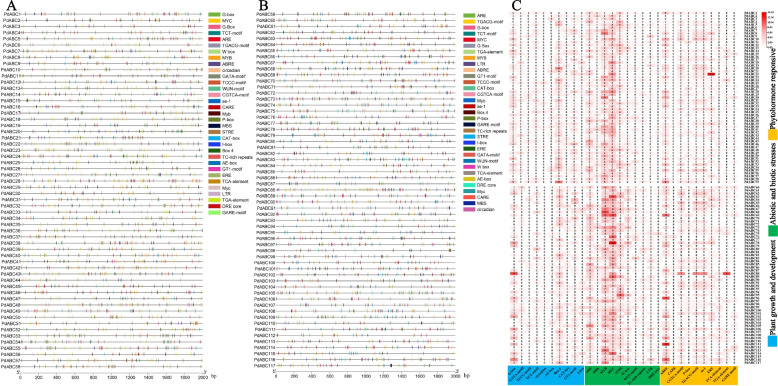
*Cis*-acting elements in the promoter regions of *PdABC* family members. **A** Promoter regions of *PdABC1* to *PdABC58*. **B** Promoter regions of *PdABC59* to *PdABC117*. Each coloured square represents a type of *cis*-acting element. **C** Number of *cis*-acting elements for *PdABC* members. The redder the box is, the more elements it contains, and the whiter the box is, the fewer elements it contains. The numbers in the boxes represent the number of *cis*-acting elements

We annotated 120 *cis*-acting elements in the 2,000 bp promoter regions of 117 *PdABC* genes, 45 of which have known functions (Table S9). In addition to basic elements such as CAAT-box and TATA-box, the promoter regions contain various light-related elements, including ACE, G-Box, and P-box. We chose to present the distribution of three functional *cis*-acting elements related to plant growth and development, abiotic and biotic stresses, and phytohormone responsiveness (Fig. 6). Plant growth and development-related elements include G-box, TCT-motif, circadian, GATA-motif, TCCC-motif, CAT-box, I-box, Box 4, AE-box, and GT1-motif elements. Abiotic and biotic stress-related elements include ARE, MBS, MYB, MYC, STRE, W box, WUN-motif, TC-rich repeats, LTR, and DRE core. Phytohormone responsiveness-related elements include ABRE, CARE, CGTCA-motif, P-box, TGACG-motif, as-1, ERE, TCA-element, TGA-element, and GARE-motif. We also counted the number of these elements in the promoter regions of each *PdABC* gene (Figure S2). The results show that *PdABC44* has the fewest elements (18), while *PdABC88* has the most elements (53). In plant growth and development, there are a total of 999 elements, with the circadian element being the least (21) and the Box 4 element being the most (254). In abiotic and biotic stresses, there are a total of 1919 elements, with the DRE core element being the least abundant (21) and the MYC element being the most abundant (505). In phytohormone responsiveness, there are a total of 1124 elements, with the CAREs being the least (10) and the ABREs being the most (272). In summary, based on the results of *cis*-acting elements, *PdABC* family members are widely involved in various biological processes.

### Expression pattern analysis of *PdABC* family members

**Fig. 7:**
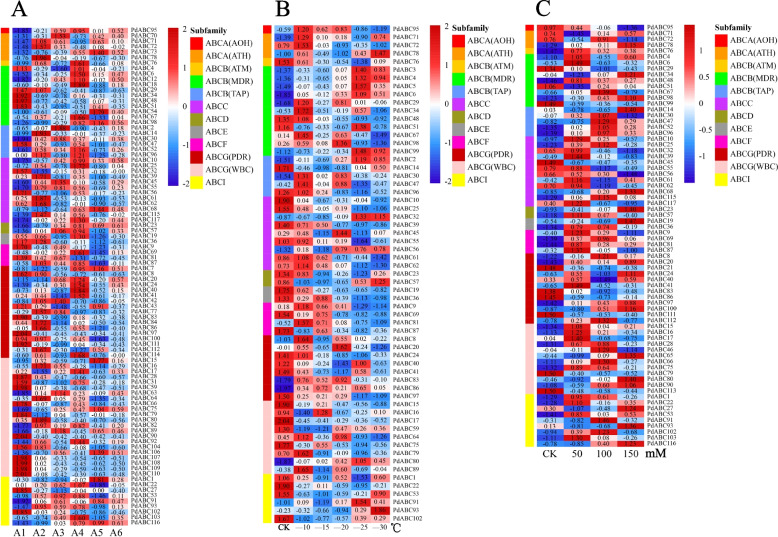
Expression patterns of *PdABC* family members. **A** Expression patterns of *PdABC* family members during the six developmental stages of ‘Wanfeng’ almond flowers. A1 (Bud stage), A2 (Enlargement stage), A3 (Inflorescence elongation stage), A4 (Small bud stage), A5 (Large bud stage), and A6 (Full blooming stage). **B** Expression patterns of *PdABC* family members in dormant one-year-old branches of ‘Wanfeng’ almond under freezing stress. **C** Expression patterns of *PdABC* family members in leaves of ‘Amanisha’ almond under salt stress. ROW normalization was used, with red squares indicating upregulation and blue squares indicating downregulation. The different colored vertical lines on the left represent different subfamilies. Numbers in the squares represent positive or negative correlation values, with values greater than or equal to 0.8 considered significant upregulation

We first removed *PdABC* family members with FPKM values consistently below 1 in all transcriptome samples (Table S10). For the six developmental stages of ‘Wanfeng’ almond flowers, we analyzed the expression patterns of 92 *PdABC* genes based on transcriptome data (Fig. 7A). The results showed that among the five stages (A1, A2, A3, A4, and A5), 30, 22, 11, 30, and 12 *PdABC* genes were significantly upregulated, respectively, whereas no significant upregulation was observed at A6 (full blooming stage). Notably, ABCG (PDR) subfamily members exhibited significant upregulation in stages A1 to A4, while ABCG (WBC) subfamily members were primarily upregulated in stage A1. Furthermore, several *PdABC* genes showed upregulation in multiple stages, such as *PdABC4*, *PdABC7*, *PdABC87*, *PdABC100*, and *PdABC104*.

For the dormant one-year-old branches of ‘Wanfeng’ almond under freezing stress, we analyzed the expression patterns of 61 *PdABC* genes based on transcriptome data (Fig. 7B). The results showed that among the CK, —10 °C, —15 °C, —20 °C, —25 °C, and —30 °C treatments, 29, 21, 2, 9, 11, and 9 *PdABC* genes were significantly upregulated, respectively. Genes from the ABCG (PDR) and ABCG (WBC) subfamilies were mainly upregulated in CK. Notably, genes from the ABCB (MDR and TAP), ABCC, ABCD, and ABCI subfamilies included *PdABC* genes upregulated at —25 °C and —30 °C. For instance, *PdABC2*, *PdABC3*, and *PdABC91*.

For salt-stressed leaves of ‘Amanisha’ almond, we analyzed the expression patterns of 72 *PdABC* genes based on transcriptome data (Fig. 7C). The results showed that among the CK, 50 mM, 100 mM, and 150 mM salt stress treatments, 11, 23, 16, and 17 *PdABC* genes were significantly upregulated, respectively. Most genes from eight subfamilies were upregulated after salt stress.

### Protein interaction and GO enrichment analysis of PdABC family members

**Fig. 8:**
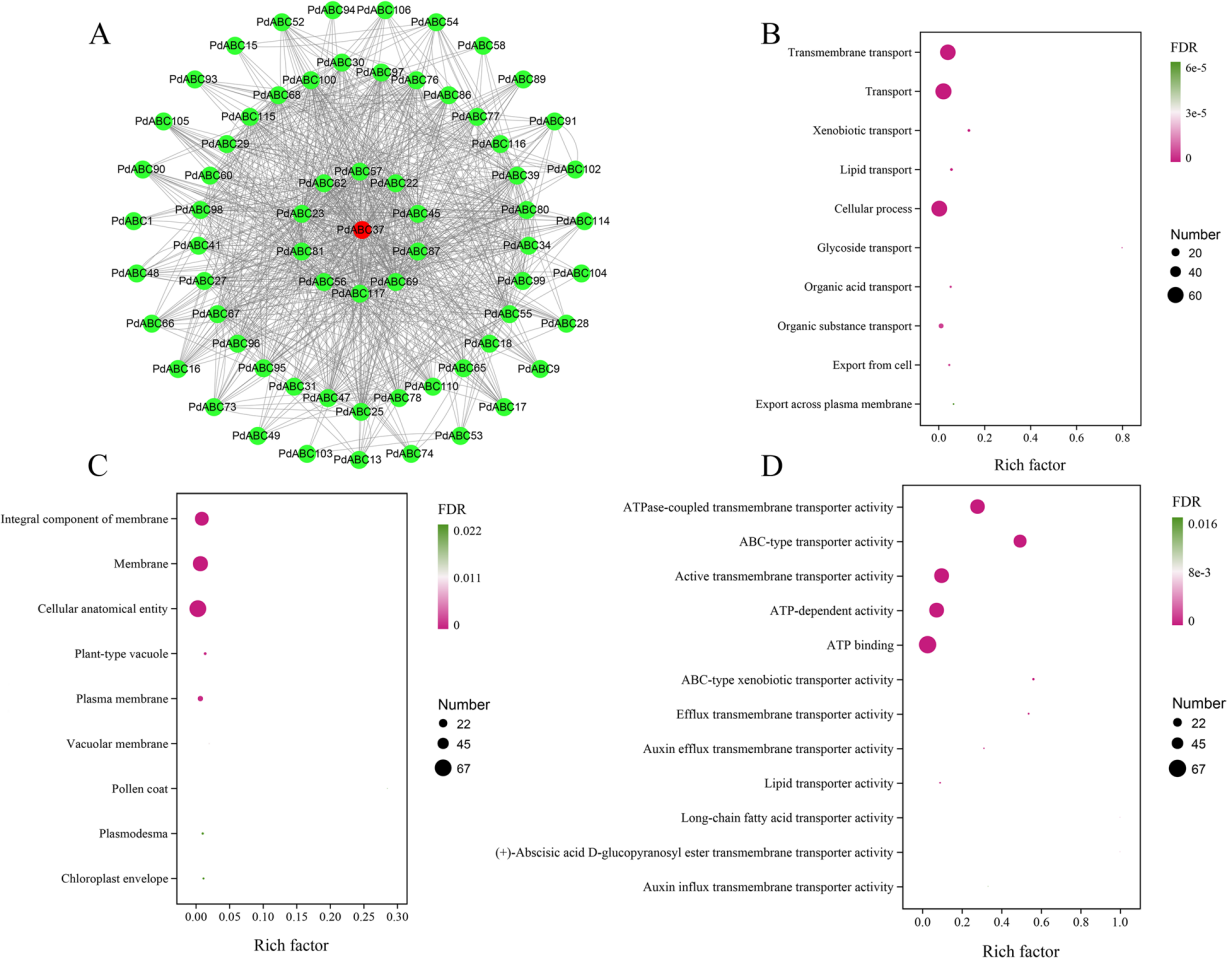
Protein interaction network and GO functional analysis of *PdABC* family members. **A** Protein interaction network of *PdABC* family members. The red font indicates hub genes. **B** Biological Process. **C** Cellular Component. **D** Molecular function

We used STRING to predict potential protein‒protein interactions among *PdABC* family members based on the *A. thaliana* database (Table S11). The results revealed that 67 PdABC family members formed a protein interaction network with 80 nodes and 460 edges. Not all nodes interacted with each other (Fig. 8A). Some proteins directly interacted with each other, such as PdABC1/PdABC22, PdABC29/PdABC100, and PdABC41/PdABC116, while others showed more complex relationships, such as PdABC45, PdABC69, and PdABC87. Additionally, *PdABC37* was predicted to be a hub gene, interacting with 41 genes. The GO enrichment analysis showed that the 67 *PdABC* genes have various protein functions (Table S12). In Biological Processes, functions such as Transmembrane Transport (GO:0055085), Transport (GO:0006810), and Cellular Processes (GO:0009987) were significant (Fig. 8B). In Cellular Components, functions including the Integral Component of Membrane (GO:0016021), Membrane (GO:0016020), and Cellular Anatomical Entity (GO:0110165) were significant (Fig. 8C). In Molecular Functions, functions such as ATPase-Coupled Transmembrane Transporter Activity (GO:0042626), ABC-Type Transporter Activity (GO:0140359), and Active Transmembrane Transporter Activity (GO:0022804) were significant (Fig. 8D).

### Fluorescence quantitative analysis of *PdABC* genes

**Fig. 9:**
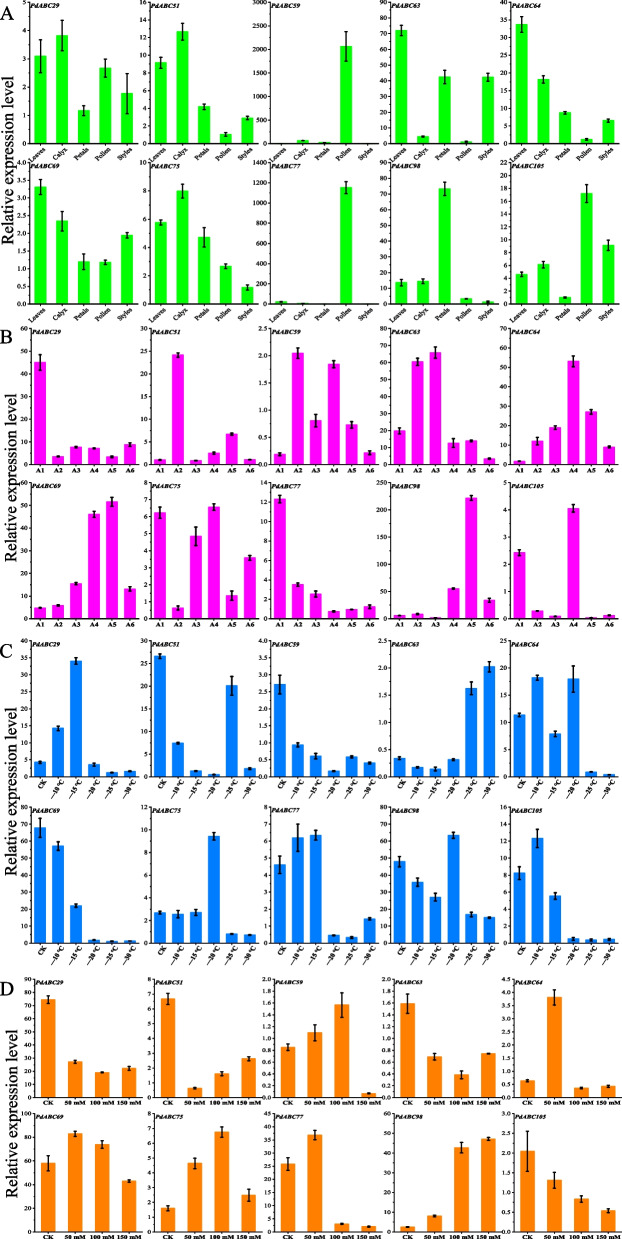
Fluorescence quantitative expression levels of 10 *PdABC* genes. **A** Expression levels in five tissues of ‘Wanfeng’ almond: leaves, sepals, petals, pollen, and pistils. **B** Expression levels at six developmental stages of ‘Wanfeng’ almond flowers: A1 (Bud stage), A2 (Enlargement stage), A3 (Inflorescence elongation stage), A4 (Small bud stage), A5 (Large bud stage), and A6 (Full blooming stage). **C** Expression levels under freezing stress at six temperature conditions in dormant one-year-old branches of ‘Wanfeng’ almond. CK: —5 °C. **D** Expression levels under four salt stress conditions in leaves of ‘Amanisha’ almond. CK: 0 mM

We conducted qRT‒PCR to examine the expression levels of 10 *PdABC* genes in various tissues of ‘Wanfeng’ almond, including leaves, sepals, petals, pollen, and pistils (Fig. 9A). The results revealed that *PdABC29*, *PdABC51*, *PdABC63*, *PdABC64*, *PdABC69*, *PdABC75*, *PdABC98*, and *PdABC105* had expression levels greater than 1 in all five tissues. Among them, *PdABC63* had the highest expression level in leaves (72.08), followed by sepals (42.39) and pistils (42.24). *PdABC64* had a relatively high expression level in leaves (33.69). *PdABC98* had the highest expression level in petals (73.23). *PdABC29*, *PdABC51*, *PdABC69*, *PdABC75*, and *PdABC105* had relatively consistent expression levels across all tissues. Notably, *PdABC59* and *PdABC77* showed exceptionally high expression levels in pollen, reaching 2063.61 and 1152.09, respectively. In summary, *PdABC* family members exhibited significant tissue-specific expression, with *PdABC59* and *PdABC77* potentially playing important roles in regulating almond pollen development and function.

For the six developmental stages of ‘Wanfeng’ almond flowers, we conducted qRT‒PCR to examine the expression levels of 10 *PdABC* genes (Fig. 9B). The results revealed that *PdABC29*, *PdABC63*, *PdABC64*, *PdABC69*, and *PdABC98* had expression levels greater than 1 across all stages. *PdABC29* had the highest expression level in stage A1 (45.00), *PdABC51* in stage A2 (24.13), and *PdABC63* had relatively high expression levels in stages A2 (60.45) and A3 (65.83). *PdABC64* and *PdABC69* had relatively high expression levels in stages A4 (53.11 and 46.04) and A5 (27.10 and 51.58). *PdABC98* had the highest expression level in stage A5 (221.84), followed by stages A4 (55.19) and A6 (34.01). Moreover, *PdABC59*, *PdABC75*, *PdABC77*, and *PdABC105* had relatively consistent expression levels across all stages, with most of their expression levels below 1. In summary, *PdABC* family members were involved in the development of ‘Wanfeng’ almond flowers and exhibited significant differential expression across different stages.

For the dormant one-year-old branches of ‘Wanfeng’ almond under freezing stress, we conducted qRT‒PCR to examine the expression levels of 10 *PdABC* genes (Fig. 9C). The results showed that *PdABC29*, *PdABC69*, and *PdABC98* had expression levels greater than 1 under all six temperature conditions. *PdABC29*, *PdABC51*, *PdABC64*, and *PdABC98* had relatively higher expression levels at specific temperature conditions. *PdABC51*, *PdABC64*, and *PdABC98* had higher expression levels at —20 °C, —25 °C, and —35 °C. In contrast, *PdABC59*, *PdABC63*, *PdABC69*, and *PdABC75* had relatively consistent expression levels across all temperatures, with most of their expression levels below 1. In summary, *PdABC* family members may be involved in the resistance of almond dormancy to freezing stress.

For salt-stressed leaves of ‘Amanisha’ almond, we conducted qRT‒PCR to examine the expression levels of 10 *PdABC* genes (Fig. 9D). The results showed that *PdABC29*, *PdABC69*, *PdABC75*, *PdABC77*, and *PdABC98* had expression levels greater than 1 in all four treatment conditions. Among them, *PdABC29*, *PdABC69*, and *PdABC77* had the highest expression levels in the CK and 50 mM treatments. *PdABC69* and *PdABC98* had higher expression levels in the 100 mM and 150 mM treatments. The remaining six genes had relatively consistent expression levels across all treatments. This result indicates that *PdABC* may improve the salt resistance of almonds, but whether it can truly improve the salt resistance of almonds needs to be verified by combining other experiments and research methods.

## Discussion

ABC transport proteins are mainly distributed in plant cell membranes, mitochondria, and vacuolar membranes, among other organelles. They play a role in the transmembrane transport of various secondary metabolites in plants, helping them adapt to external environmental stresses [45]. To date, ABC transport proteins have been identified and studied in several plant species. For example, *A. thaliana* (129), *O. sativa* (123) [46], *Zea mays* (133) [47], *Glycine max* (261) [48], and *Camellia sinensis* (170) [49], among others. In the family *Rosaceae*, ABC transport protein members have been identified in various species, such as *M. domestica* (191), *Rubus occidentalis* (98), and *Fragaria vesca* (115) [30, 50].

The number of ABC transport proteins in plants is not strictly related to the size of their genomes. In ‘Wanfeng’ almond, we identified 117 *PdABC* genes, which is similar to the number in *P. avium* (118) but significantly different from that in *P. persica* (138) and *P. mume* (162) [50, 51]. *PdABC* members exhibit considerable variation in their physicochemical properties, such as amino acid number, molecular weight, and isoelectric point. Subcellular localization prediction suggests that over half (79) of the members are localized to the plasma membrane, consistent with the characteristics of *ABC* members in species such as *M. domestica* and *C. sinensis*. Based on the subfamily classification of *A. thaliana ABC* members, we divided *PdABC* members into eight subfamilies: ABCA (AOH and ATH), ABCB (ATM, TAP, and MDR), ABCC, ABCD, ABCE, ABCF, ABCG (PDR and WBC), and ABCI. The distribution of *ABC* numbers in these subfamilies among different species exhibits a high degree of conservation (see Table S4). Notably, the proportion of *PdABC* members in the ABCG subfamily is significantly higher than that in *P. avium*, *P. mume*, and *P. persica*. This difference may be due to segmental duplication and tandem repeat amplification, increasing the number of *PdABC* members in the ABCG subfamily. Additionally, based on clustering results with *ABC* genes associated with floral organ functions in five species, we predicted that PdABC members may regulate floral organ functions. This provides valuable insights for further research on almond floral organ development and self-incompatibility (see Fig. 1B).

Gene function is closely related to the presence of conserved motifs in protein sequences [52]. All ten motifs identified in the protein sequences of *PdABC* members are characteristic motifs of ABC transport proteins. Members within the same subfamily exhibit more conserved motif types, suggesting a higher level of conservation among *PdABC* members within the same subfamily. This result aligns with the findings of conserved motif analysis in *ABC* family members in other plant species [50]. Gene structure information can provide clues about the evolution of gene family members [52]. In the *PdABC* family, six members have no introns, and the number of exons and introns varies significantly, regardless of whether the members belong to the same subfamily. Only members clustered in the same branch exhibited a similar number of exons and introns. This result is consistent with the gene structure features of *ABC* family members in *C*. *sinensis*. In summary, the conserved motifs within members of the same subfamily and the differences in exon and intron numbers may be due to functional differentiation during gene evolution.

Almond and four other *Prunus* fruit trees, including *P. persica*, *P. avium*, and *P. mume*, exhibit an uneven distribution of *ABC* members across their eight chromosomes. This nonuniform distribution is likely a result of different gene duplication events on the chromosomes [50, 51]. Notably, 43 *PdABC* genes are located in regions of low gene density on the chromosomes, suggesting that these genes may serve crucial functions in the low gene density regions. Gene duplication is a major factor contributing to the expansion and evolution of gene family members, aiding species in adapting to environmental changes and maintaining vital life processes [53]. In this study, we identified 12 pairs of segmental duplications and 11 pairs of tandem duplicated genes within the *PdABC* family. The total number of duplicated *PdABC* genes is similar to that of *P. persica* (26) and *C. sinensis* (23) but lower than that of *P. avium* (47), *P. mume* (39), *Linum usitatissimum* (88) and other species [54]. Furthermore, there were 32, 0, 108, and 94 pairs of collinear genes between almond and *A. thaliana*, *O. sativa*, *M. domestica*, and *P. persica*, respectively. Specifically, the one-to-one relationship between almond and *P. persica* in terms of *ABC* collinear genes may result from the limited degree of evolutionary divergence between these two species, suggesting that their *ABC* genes may share similar functions. Ka/Ks values serve as an effective measure for studying the evolution and selection of duplicated genes [55]. The Ka/Ks values for duplicated *PdABC* genes are all less than 1, indicating that these duplicated genes have mainly undergone purifying selection, emphasizing their highly conserved functions. Similarly, the Ka/Ks values for collinear gene pairs between different species were all less than 1, indicating that the evolution of *ABC* genes in different species is under strong purifying selection pressure, suggesting that they may have similar or related gene functions.

*Cis*-regulatory elements in promoter regions can regulate gene expression [56]. The results of upstream *cis*-regulatory elements suggest that *PdABC* genes are involved in various functions, such as growth and development, stress responses, and hormone regulation in ‘Wanfeng’ almond. Investigating gene expression during tissue development and under adverse environmental conditions is essential for understanding the molecular mechanisms of biological development [57]. Based on transcriptome data, we explored changes in FPKM values for *PdABC* family members in leaf tissues of ‘Wanfeng’ almond at six stages of flower development, under six freezing stress treatments of one-year-old dormant branches, and under four different salt concentration stress treatments in ‘Amanisha’ almond leaves. In all different treatment samples, several *PdABC* family members showed upregulated gene expression. Many *PdABC* family members exhibited significant upregulation during three stages of flower development: sprouting, enlargement, and small flower bud formation. They also displayed significant expression under both control and 10 °C temperatures, with a decrease in gene expression as the temperature decreased further, and their expression was also altered under salt stress conditions. Overall, these results indicate that *PdABC* family members likely play a role in regulating the growth and development of various almond tissues and enhancing almond tolerance to abiotic stress. However, further in-depth research is needed to confirm their specific functions and regulatory mechanisms. Protein‒protein interactions are important in the study of species traits [58]. We predicted potential protein‒protein interactions among *PdABC* members. A protein‒protein interaction network composed of 67 *PdABC* members, with 80 nodes in total, was predicted. *PdABC37* was predicted as a hub gene in this network, which can provide insights into the relationships among *PdABC* members for future research.

Research on the relationship between *ABC* genes and plant floral organ function has yielded important results [9]. For instance, *OsMRP15* and *VvABCC1* are involved in anthocyanin transport in *O. sativa* and *M. truncatula*, respectively [22, 59]. *PhABCG1* is involved in the release of floral fragrance compounds in *Clarkia breweri* [28]. In our study, we used fluorescence quantification to examine the expression levels of 10 *PdABC* genes in five different tissues (leaves, sepals, petals, pollen, and pistils) and during six stages of flower development in ‘Wanfeng’ almond. The expression of these genes exhibited significant differences among the tissues and developmental stages. For example, *ATABCB9* and *ATABCB31* are known to promote the transport of sterol glycosides on the surface of *Arabidopsis* pollen, affecting pollen wall maturation and growth [60]. Similarly, *PdABC59* and *PdABC77* displayed high expression levels in pollen, clustering with *ATABCB9* and *ATABCB31*, indicating their potential importance in regulating almond pollen development. Moreover, *MdABCF* is involved in the transfer of apple *S*-RNase to the pollen tube, leading to self-incompatibility [61]. *PdABC69*, which exhibits high conservation in clustering with *MdABCF*, was significantly expressed in both pollen and pistils, making it a candidate reference gene for studying self-incompatibility in almond. *ATABCB13* and *ATABCB15* are known to regulate the lipid substances necessary for petal epidermal cell cuticle formation in *Arabidopsis*, influencing petal development [62, 63]. In our study, *PdABC63* and *PdABC64* had high expression levels in petals and clustered with *ATABCB13* and *ATABCB15*, particularly during the swelling, flower bud elongation, small flower bud, and large flower bud stages. We speculate that these two genes might play roles in almond petal development and flower morphology formation. Additionally, *PdABC98* may also be involved in regulating almond petal growth and development. *PdABC69* and *PdABC98* exhibited higher expression levels during the small and large flower bud stages, suggesting their involvement in late-stage flower organ development and petal opening. In summary, *PdABC* family members likely play crucial roles in various stages of almond flower development and flower organ development, providing insights for future research in almond-related studies.

While extensive research has been conducted on the relationship between *ABC* genes and plant responses to abiotic stressors such as water, heavy metals, and ions [64], studies on temperature stress are more prevalent, with cold stress research mainly focused on *C. sinensis,* and studies on freezing stress are lacking. In our study, fluorescence quantification revealed that 10 *PdABC* genes showed significantly higher expression levels at one or more temperatures following freezing stress treatments on one-year-old dormant branches of ‘Wanfeng’ almond. These results can provide a reference for researching the response mechanisms of *ABC* genes to freezing stress in other plants. Notably, *PdABC98* showed expression levels exceeding 10 at all six temperatures, with a particularly high expression level at —20 °C, suggesting a potential role for this gene in protecting almond during the dormancy period from freezing stress. Research has elucidated the role of *ABC* genes in plant responses to salt stress [65, 66]. In our study, fluorescence quantification revealed differential expression levels of 10 *PdABC* genes in ‘Amanisha’ almond under four different salt concentration stress treatments. *PdABC29*, *PdABC69*, and *PdABC98* displayed relatively high expression levels under all four salt stress conditions, suggesting a possible role in almond’s response to salt stress. Conversely, *PdABC59*, *PdABC63*, and *PdABC105* exhibited lower expression levels. This result shows that the expression levels of *PdABC* members change under different concentrations of salt stress. However, whether *PdABC* genes can improve the salt resistance of almonds requires further in-depth functional verification.

## Conclusion

In this study, we identified 117 *PdABC* genes in the whole genome of ‘Wanfeng’ almond and conducted analyses on the protein characteristics, gene structure, gene duplication, and evolutionary aspects of the *PdABC* gene family. We classified the *PdABC* family members into 8 subfamilies, with the highest number of gene duplications occurring in the ABCG subfamily. Members of the same subfamily have high protein conservation motif types but have large differences in protein length and exon number. Gene mapping showed that *PdABC* members were unevenly distributed on the 8 chromosomes. The interspecies collinearity results indicate that *ABC* members of almond and peach have higher conservation. The promoter results indicate that *PdABC* members are widely involved in various functions, such as ‘Wanfeng’ almond growth and development, stress response, and hormone regulation. Expression patterns indicate that *PdABC* members are involved in almond flower development in response to freezing and salt stress. Using fluorescence quantification, we explored the expression levels of 10 *PdABC* genes in various almond tissues, including flowers during developmental stages, one-year-old dormant branches under freezing stress, and leaves under salt stress. These findings provide valuable resources for understanding the functions and molecular mechanisms of *PdABC* family members in almond.

### Supplementary Information

**Additional file 1: Table S1.** Sequences of primers for qRT-PCR. **Table S2.** Fluorescence quantitative expression level statistics of 10 *PdABC* genes. **Table S3.** Characteristics of *PdABC* gene family members in almond genome. **Table S4.** Statistics of *ABC* gene family members in 9 species. **Table S5.** Prediction of functional genes for floral organ development of *PdABC* family members. **Table S6.** Gene density of each chromosome of the almond genome. **Table S7.** *PdABC* genes replication and homologous gene. **Table S8.** *PdABC* genes replication and homologous gene Ka/Ks values. **Table S9.** *Cis*-element analyses of the *PdABC* genes. **Table S10.** *PdABC* family member FPKM value. **Table S11.** String interactions. **Table S12.** Results of GO enrichment annotation for PdABC protein interaction members.

**Additional file 2: Figure S1. **LOGO map corresponding to 10 Motif sequences. **Figure S2. **Statistics on the number of *cis-*acting elements in the *PdABC *family, including Plant growth and development, Abiotic and biological stresses, and Phytohormone response. (A) Statistics on the number of three functional *cis-*acting elements in *PdABC1 *~ *PdABC58 *members. (B) Statistics on the number of three functional *cis-*acting elements in *PdABC59 *~ *PdABC117 *members. (C) Total number of *cis-*acting elements for each type.

## Data Availability

The transcriptome data are owned by our team and can be accessed through the CNCB database (https://www.cncb.ac.cn/). ‘Wanfeng’ almond six flower developmental stages (GSA Number: CRA007615), a gradient of six freezing temperature stress conditions for dormancy (GSA Number: CRA007323), and salt stress treatments for ‘Amanisha’ almond (GSA Number: CRA013033).
