# Supplementary material for: Genome-wide analysis of the ABC gene family in almond and functional predictions during flower development, freezing stress, and salt stress
Source: BMC Plant Biol. 2024 Jan 2;24:12. doi: 10.1186/s12870-023-04698-7 (PMC10759767; doi:10.1186/s12870-023-04698-7)
Supplement: Supplementary file 2 — Additional file 2: Figure S1. LOGO map corresponding to 10 Motif sequences. Figure S2. Statistics on the number of cis-acting elements in the PdABC family, including Plant growth and development, Abiotic and biological stresses, and Phytohormone response. (A) Statistics on the number of three functional cis-acting elements in PdABC1 ~ PdABC58 members. (B) Statistics on the number of three functional cis-acting elements in PdABC59 ~ PdABC117 members. (C) Total number of cis-acting elements for each type. [file 12870_2023_4698_MOESM2_ESM.docx]

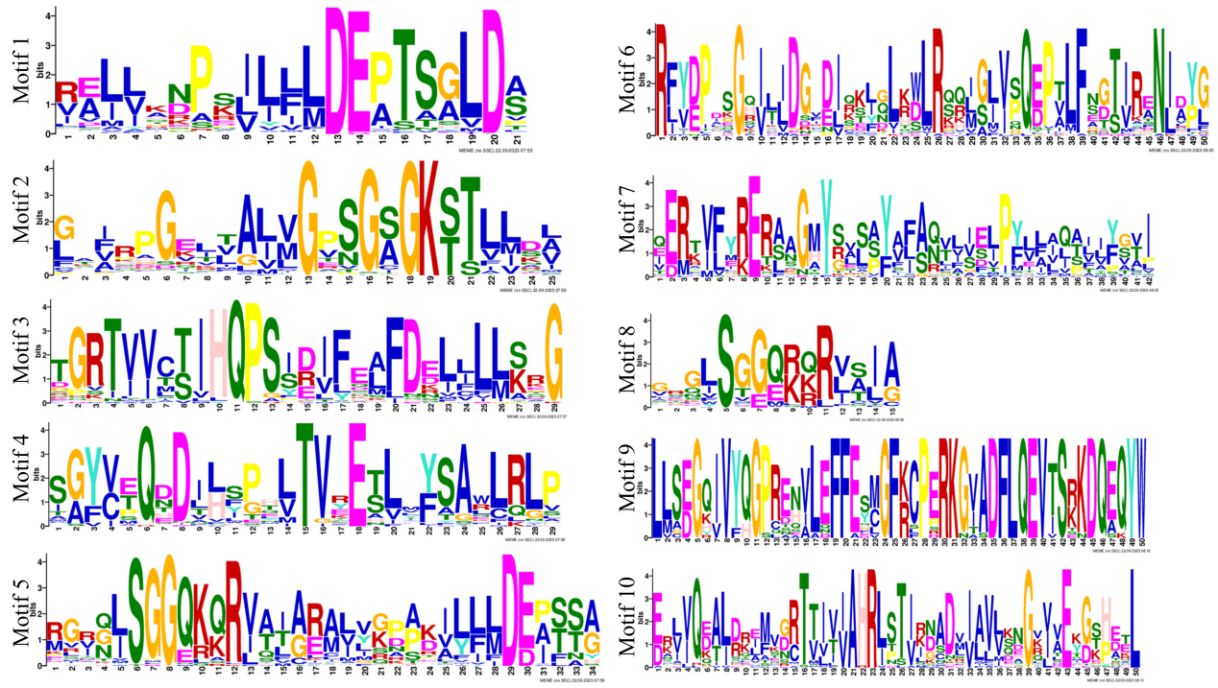


**Figure S1:** LOGO map corresponding to 10 Motif sequences


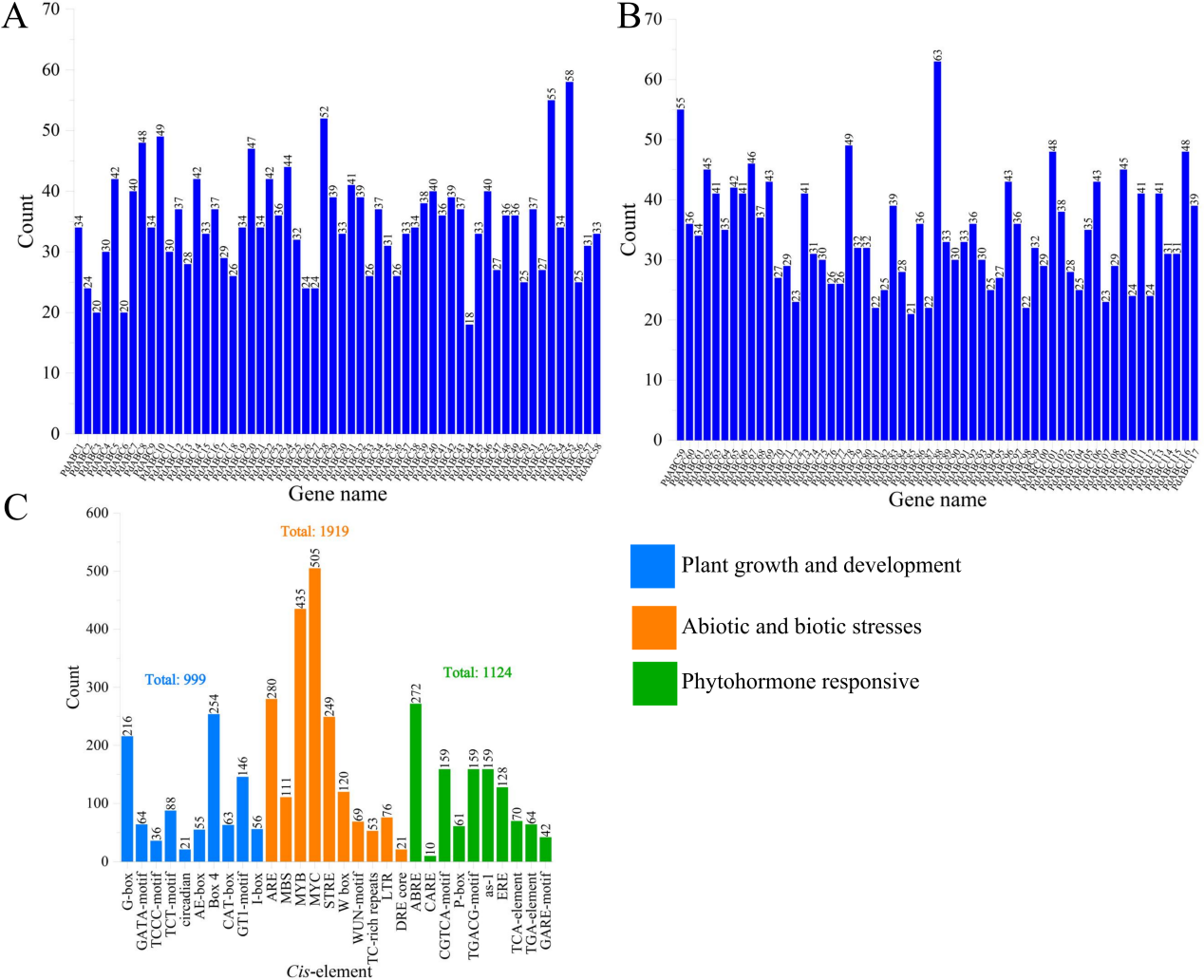


**Figure S2:** Statistics on the number of *cis-*acting elements in the *PdABC* family, including Plant growth and development, Abiotic and biological stresses, and Phytohormone response. (A) Statistics on the number of three functional *cis-*acting elements in *PdABC1* ~ *PdABC58* members. (B) Statistics on the number of three functional *cis-*acting elements in *PdABC59* ~ *PdABC117* members. (C) Total number of *cis-*acting elements for each type.
